# Effect of Nitric Oxide on Basolateral Amygdala on Persistence of Anxiety and Depression in Stressed Male Rats

**DOI:** 10.32598/bcn.9.10.100

**Published:** 2019-01-01

**Authors:** Esmaeil Nikkar, Hassan Ghoshooni, Mohammad Mahdi Hadipour, Hedayat Sahraei

**Affiliations:** 1. Department of Physiology and Biophysics, School of Medicine, Baqiyatallah University of Medical Sciences, Tehran, Iran.; 2. Neuroscience Research Center, Baqiyatallah University of Medical Sciences, Tehran, Iran.

**Keywords:** Anxiety, Basolateral Amygdala, Depression, Nitric Oxide, L-Arginine, Stress

## Abstract

**Introduction:**

The current study aimed at investigating the role of Nitric Oxide (NO) in the maintenance of anxiety and depression induced by stress in male Wistar rats using intra-Basolateral Amygdala (BLA) injection of NO precursor, L-arginine, Nitric Oxide Synthase (NOS) inhibitor, and L-NAME.

**Methods:**

Two 23-gauge stainless steel cannulas were placed in the BLA, stereotaxically. Seven days later, animals experienced electro foot shock stress based on the following protocol: animals experienced four sessions of stress for 60 minutes in four consecutive days. Five minutes before each stress session, the animals received different doses of L-arginine or L-NAME (1, 5 and, 10 μg/rat) or saline (0.5 μL/rat) intra-BLA. Six days after the stress termination, animals were tested for maintenance of anxiety-like behavior (elevated plus maze; EPM) and eight days after the stress they were examined for depression (forced swimming test; FST).

**Results:**

Stress reduced the time and number of open arms and decreased motor activity on EPM. Stress-induced anxiety was inhibited by L-arginine and L-NAME (1, 5, and 10 μg/rat). L-Arginine and L-NAME induced anxiety in non-stressed rats. Stress also increased the immobility time in animals in FST paradigm. Interestingly, both L-arginine and L-NAME, in all doses reduced the stress effect.

**Conclusion:**

BLA nitric oxide may play a pivotal role in anxiety and depression induced by stress in rats. Since the effects of both L-arginine and L-NAME were similar, NO might have a modulatory role in the BLA.

## Highlights

- Intra-basolateral amygdala NOergic agents could induce anxiety and depression in healthy animals.
- Intra-basolateral amygdala NOergic agents reduce stress-induced anxiety and depression.

## Plain Language Summary

All of us experience unpleasant events in our life in which we sense helpless, afflicted, and misery. These terms in fact may be the signs of what we call anxiety or depression. Brain plays a major role in initiation and continuation of these moods. Anxiety and depression treatment is still inefficient because nobody knows exactly how the brain compartments are affected by anxiety and depression. Anxiety and depression can be induced by stress and in animal model. We induced anxiety and depression in the rats and then tried to activate a neurotransmitter system called nitric oxide or NO in the important part of amygdala (basolateral amygdala [BLA]) for further evaluation and understanding the role of this brain system in the anxiety and depression. We found that if the NO were activated by itself, it might lead to anxiety, and depression. Thus, NO can regulate the BLA so that it could overcome the regular changes leading to anxiety and depression. However, when the system get activated in the stressed animals, it could effectively reduce stress effect. A surprising finding that indicated the role of NO in BLA for regulation of stress side effects.

## 1. Introduction

Anxiety and depression are among the most important complications of stressful events. Various brain structures are involved in the development of these mental disorders. Glucocorticoid hormones released during stress e responsible elements for the changes occurred after stress full events (Miller & O’Callaghan, 2002). Eevidence suggests that hippo-campus and amygdala are among the brain structures that play a role in mediation of stress-induced anxiety and depression (Vyas, Mitra, Shankaranarayana Rao, & Chattarji, 2002). Contrary to the hippocampus, the dendritic structures and neurons in the amygdala, especially Basolateral Amygdala (BLA), show hypertrophy in response to the stress (Roozendaal, McEwen, & Chattarji, 2009; Mitra, Jadhav, McEwen, Vyas, & Chattarji, 2005).

Immunehistochemycal studies indicate that the BLA neurons receive glutamate input from different brain regions including frontal cortex and striatum (Pape & Pare, 2010). It should also be noted that neurons in this region express high levels of Nitric Oxide Synthase (NOS) enzyme activated during the glutamate neurotransmission (Schafe et al., 2005). There are three different forms of the NOS namely NOS1 or neuronal NOS, NOS2, or inducible NOS, and NOS3 or endothelial NOS (Gathwaite & Boulton, 1995). These enzymes are activated by Ca^+2^ ions entry into the neuron during the N-Methyl-D-Aspartate (NMDA) receptor activity and produce NO from L-arginine (Gathwaite & Boulton, 1995). NO can stimulate glutamate, dopamine, acetylcholine, serotonin, and Gamma-Amino-Butyric-Acid (GABA) (Trabace & Kendrick, 2000) released from their corresponding neurons in various brain regions including BLA and hippocampus. On the other hand, the role of NO in anxiety and depression is well established (Pitsikas, 2018).

In this regard, it is observed that systemic L-NAME administration reduces the anxiety evaluated by EPM in the rats (Faria et al., 1997). In addition, it is clear that intra-dorsal raphe nucleus administration of L-arginine reduces the anxiety in EPM method in the rats, whereas intra-dorsal raphe nucleus injections of L-NAME and 7-nitroindazole induce anxiety in the rats (Spiacci, Kanamaru, Guimaraes, & Oliveira, 2008). Thus, there might be a complex interaction between NO and stress-induced anxiety. Since the role of NO in the BLA is not investigated in this regard, by injection of L-arginine as a precursor of NO synthesis and L-NAME as an inhibitor of NOS in the BLA, the current study aimed at evaluating the role of NO to maintain stress-induced anxiety and depression in stressed rats.

## 2. Methods

### 2.1. Animals

Male Wistar rats (Weighing 200–250 g) purchased from Pasture Institute (Tehran, Iran) were used in the current study. The animals were transferred to the animal room at the Neuroscience Research Center of Baqiyatallah University of Medical Sciences and kept in cages (four animals per cage) at controlled temperature conditions (22±2°C) with free access to the standard rat chow and tab water ad lib. Each animal (n=6 per group) was used once.

### 2.2. Drugs

Ketamine hydrochloride (alpha-san-Holland), xylazine hydrochloride (Sigma-USA), L-arginine (Merck-Germany), and NG-nitro-L-arginine methyl-ester (L-NAME; Sigma-USA) were used in the study. All drugs were dissolved in the sterile saline in a volume of 1 mL/kg (ketamine and xylazine) or 0.5L/rat (L-arginine and L-NAME).

### 2.3. Evaluation of anxiety (Elevated Plus Maze)

Elevated Plus Maze (EPM) was used to evaluate anxiety in the animals. The apparatus was described elsewhere (Roohbakhsh, Moghaddam, Massoudi, & Zarrindast, 2007). Briefly, it consisted of four crossed arms made from Plexiglas. The size of the arms was 100×10 cm. Two arms had no walls (open arms) and two arms had a 50×50 cm wall (closed arms). The arms were separated by a central square (10×10 cm) platform. Each EPM experiment session lasted five minutes. The test was initiated by placing the rat on the central platform of the maze, facing one of the closed arms.

Animals’ position and movement were recorded and analyzed on line using a video tracking system (Borj-e-Sanat Co., Tehran, Iran). Four parameters were recorded in this test: the frequency of rat entry into the open arms, the frequency of rat entry into the closed arms, the total amount of time the rats spent in the open arms, and the total amount of time the rats spent in the closed arms. For each animal, Open Arms Entries (OAE%) and Open Arms Time (OAT%) were calculated as follows:

$$\begin{array}{l} \begin{array}{l} \mathrm{The}\;\mathrm{percentage}\;\mathrm{entries}\;\mathrm{on}\;\mathrm{open}\;\mathrm{arms}=(\mathrm{number}\;\mathrm{of}\\ \mathrm{entrance}\;\mathrm{to}\;\mathrm{open}\;\mathrm{arm}/\mathrm{number}\;\mathrm{of}\;\mathrm{entrance}\;\mathrm{to}\;\mathrm{closed}\\ \mathrm{arm}+\mathrm{number}\;\mathrm{of}\;\mathrm{entrance}\;\mathrm{to}\;\mathrm{open}\;\mathrm{arm})\times 100 \end{array} \end{array}$$

$$\begin{array}{l} \begin{array}{l} \mathrm{The}\;\mathrm{percentage}\;\mathrm{spent}\;\mathrm{in}\;\mathrm{open}\;\mathrm{arms}=(\mathrm{time}\;\mathrm{spent}\;\mathrm{on}\\ \mathrm{open}\;\mathrm{arm}/\mathrm{time}\;\mathrm{spent}\;\mathrm{on}\;\mathrm{open}\;\mathrm{arm}+\mathrm{time}\;\mathrm{spent}\;\mathrm{on}\;\mathrm{closed}\\ \mathrm{arm})\times 100 \end{array} \end{array}$$

Animals’ motor activity was calculated as follows:

$$\begin{array}{l} \begin{array}{l} \mathrm{Motor}\;\mathrm{activity}=\mathrm{number}\;\mathrm{of}\;\mathrm{open}\;\mathrm{arm}\;\mathrm{entries}+\mathrm{number}\\ \mathrm{of}\;\mathrm{closed}\;\mathrm{arm}\;\mathrm{entries}. \end{array} \end{array}$$

Significant increase in OAT% and OAT% indicated anxiety reduction in this test.

### 2.4. Forced swimming test

This test was used to induce depression after a severe stress experience in rodents (Porsolt, Le Pichon, & Jalfre, 1977). For this propose, a Plexiglas container 50 cm high × 30 cm in diameter filled up to 20 cm with 25°C water was used. Each rat was gently dropped in the container from 20 cm above. Immobilization of the animal was considered as the start of depression (Porsollt et al., 1977).

The experiments were performed from 9 AM to 4 PM. The experimental conditions were similar for all animals. Forced Swimming Test (FST) lasted six minutes. In this test, initial first two minutes were considered as adaptation time, and the activities of the animals were not recorded. The activities of the animals in the next four minutes were recorded and immobility time for each animal was calculated using offline video type (Porsollt et al., 1977).

### 2.5 Surgical procedures

Surgery was performed under ketamine hydrochloride (100 mg/kg) plus xylazine (8 mg/kg) anesthesia. Two 23-gauge stainless steel guide cannulas were implanted bilaterally 500 μm above the injection site using the Paxinos and Watson atlas for rat (Paxinos & Watson, 2007). The BLA coordinates according to this atlas were: −3.3 mm (incisor bar), −2.8 mm (AP), ±5 mm (L), and 6.8 mm (D) from the skull surface. The cannulas were secured using two stainless steel screws and dental acrylic. Then, two dummy cannulas (stainless steel wire 0.1 mm diameter) were inserted into the guide cannulas. These dummy cannulas remained in the guide cannulas until injections. The length of the dummy cannulas was matched with that of the guide cannulas. The ani mals were allowed seven days to recover from the side effects of surgery and anesthesia.

Drugs injections were performed as follows: each animal was restrained; dummy cannulas were removed and two 30-gauge injection cannulas that their tips were 500 μm bellow the tip of the guide cannulas were inserted into the guide cannulas. L-arginine and L-NAME solutions were slowly injected into the BLA in a total volume of 0.5 μL/rat (0.25 μL in each side) over a period of 60 seconds. The injection cannulas were left in the guide cannulas for an additional 60 seconds to facilitate drug diffusion.

### 2.6. Experimental design

#### 2.6.1. The effect of intra-BLA NOergic drugs injections on the maintenance of anxiety in the stressed rats

Sixteen groups of the animals were selected for these experiments (n=6–9/group). Six groups received different doses of L-arginine or L-NAME (1, 5, and 10 μg/rat) in their BLA in the four consecutive days and five minutes before they were placed in the communication box in its off state. Six other groups received the same doses of L-arginine or L-NAME. Five minutes later, they were placed in the apparatus and experienced electric foot shock. Control groups received saline. These animals were tested for anxiety six days later.

#### 2.6.2. Effect of intra-BLA L-arginine and L-NAME administrations on maintenance of depression in the stressed animals

Two days after the anxiety test (i.e. 8^th^ day of the experiment), maintenance of depression-like behavior was tested in the animals (same 16 groups).

### 2.7. Histology

**Figure 1.**
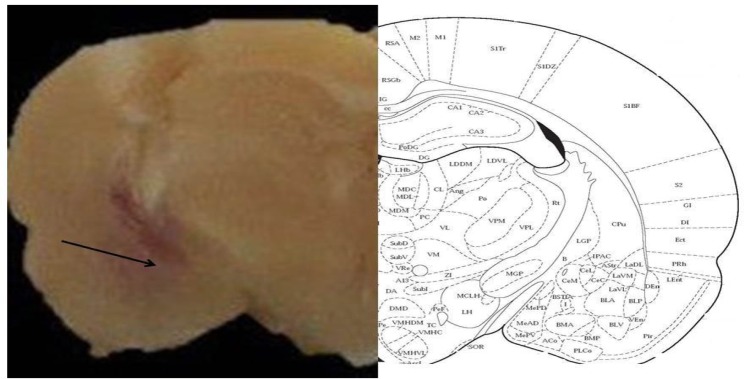
The site of injection of L-arginine and L-NAME in the Basolateral Amygdala (BLA)

After completion of the experiments, the animals were deeply anesthetized with high doses of ketamine and a methylene blue 4% solution was injected into the guide cannula at a volume of 0.25 μL/side. The animals’ brain was removed surgically after infusion of transcardiac saline and 4% formalin. The brains were dissected and the site of injection was determined by a professional anatomist. Only the results of those animals that their injections were correctly performed were considered for further statistical evaluations (Figure 1).

### 2.8. Statistical analysis

Data were expressed as mean±Standard Error of Mean (Mean±SEM) of variables. Two-way ANOVA was used to analyze the data using stress and drug as factors followed by Tukey post hoc test. In all of the analysis, P<0.05 was considered statistically significant.

## 3. Results

### 3.1. The effect of L-arginine on the maintenance of anxiety in stressed rats

**Figure 2.**
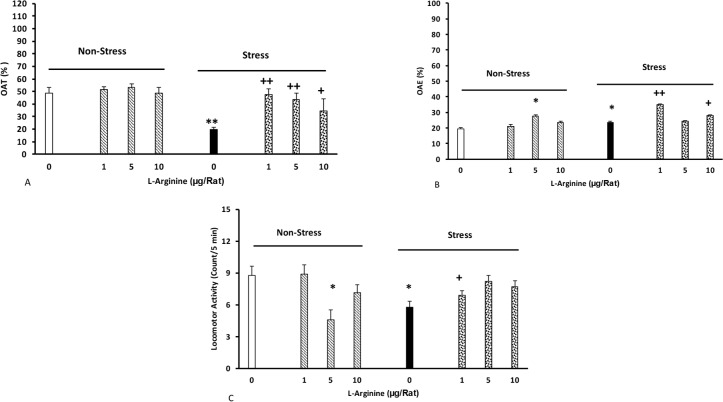
The effect of L-arginine on the anxiety induced by stress Effect of intra-BLA injections of L-arginine on the percentage of elapsed time (A), percentage of entrance (B), and distance traveled (C) by the rats Animals first received different doses of L-arginine (1, 5, and 10 μg/rat) or saline five minutes before induction of stress for four consecutive days Six days later, their anxiety was recorded in the plus maze for five minutes (n=6–8/group). * P<0.05, ** P<0.01 different from the control group. +P<0.05, and ++P<0.01 different from the stress group.

The effect of L-arginine on the anxiety induced by stress is shown in Figure 2 (A–C). Different doses of L-arginine (1, 5, and 10 μg/rat) were injected to the animals’ BLA five minutes before each stress sessions. The other groups received the same doses of the drug, but did not experience stress. Control groups received saline (0.5 μ/rat).

The results showed that stress reduced the amount of time in the open arm, and motor activity, but increased the number of open arm entries. In addition, L-arginine injections inhibited the effects of stress on time [twoway ANOVA, intragroup comparison: L-arginine effect: F(7,45)=2.22, P<0.01, stress effect: F(1,45)=3.6, P<0.01, L-arginine vs. stress: F(7,45)=3.32, P<0.01], but increased the number of open arm entries [two way analysis, intragroup comparisons: L-arginine: F(7,45)=3.12, P<0.01, stress: F(1,45)=2.84, P<0.01, L-arginine vs. stress: F(7,45)=2.98, P<0.01], and decreased motor activity [two-way analysis, intragroup: L-arginine: F(7,45)=2.74, P<0.01, stress: F(1,45)=3.14, P<0.01, L-arginine vs. stress: F(7,45)=4.21, P<0.01], (Figure 2 A–C).

### 3.2. The effect of L-NAME on the maintenance of anxiety in stressed rats

**Figure 3.**
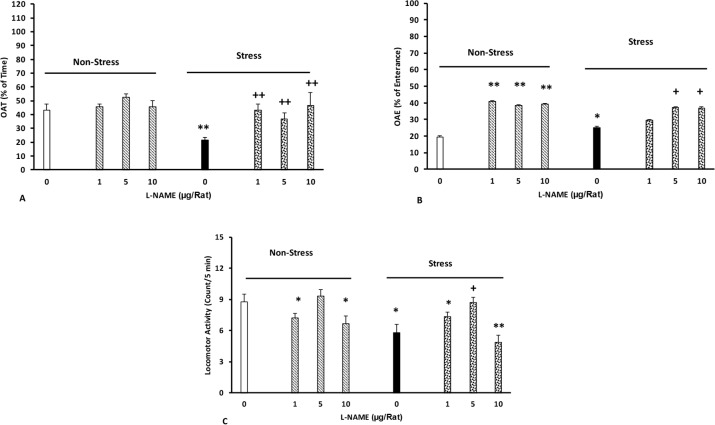
Effect of intra-BLA injections of L-NAME on the percentage of elapsed time (A), percentage of entrance (B), and distance traveled (C) by the rats Animals first received different doses of L-NAME (1, 5, and 10 μg/rat) or saline five minutes before induction of stress for four consecutive days. Six days later, their anxiety was recorded in the plus maze for five minutes (n=6–8/group). *P<0.05 and **P<0.01 different from the control group.+P<0.05 and ++P<0.01 different from the stress group.

The current study data indicated that L-NAME injection inhibited the effects of stress on time [two-way analysis, intragroup: L-NAME: F(7,45)=2.16, P<0.01, stress: F(1,45)=2.71, P<0.01, L-NAME vs. stress: F(7,45)=2.51, P<0.01], number of open arm entries [two-way ANOVA, intragroup: L-NAME: F(7,45)=2.67, P<0.01, stress: F(1,45)=3.01, P<0.01, L-NAME vs. stress: F(7,45)=3.25, P<0.01], and locomotor activity [two-way analysis, intragroup: L-NAME: F(7,45)=2.78, P<0.01, stress: F(1,45)=3.2, P<0.01, L-NAME vs. stress: F(7,45)=2.63, P<0.01]. However, in non-stressed animals, L-NAME increased the number of open arm entries and reduced motor activity in the animals (Figure 3 A–C).

### 3.3. The effect of L-arginine on maintenance of depression in stressed animals

**Figure 4.**
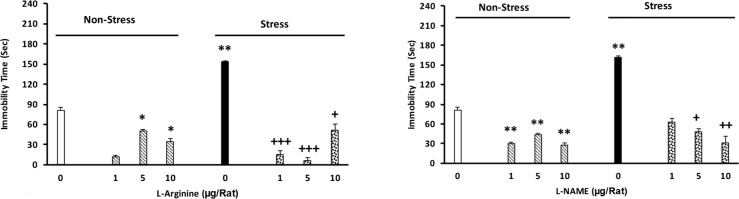
Effect of intra-BLA injections of L-arginine (A) and L-NAME (B) on immobility induced by forced swimming in the rats Animals first received different doses of L-arginine or L-NAME (1, 5, and 10 μg/rat) or saline five minutes before induction of stress for four consecutive days; Eight days later, their motor activity was recorded in the forced swimming paradigm for four minutes (n=6–8/group). *P<0.05, **P<0.01, different from the control group.+P<0.05, ++P<0.01, and +++P<0.001 different from the stress group.

The results obtained in this section showed that stress could significantly increase immobilization time in the animals. Infusion of L-arginine reduces the stress effects. L-arginine increases the mobility in non-stressed animals [two-way analysis, intragroup: L-arginine: F(7,45)=3.8, P<0.001, stress: F(1,45)=4.12, P<0.001, L-arginine vs. stress: F(7,45)=3.41, P<0.001], (Figure 4A).

### 3.4. The effect of L-NAME on the maintenance of depression in the stressed animals

Significant increases in the immobility time in the stressed animals were observed, while L-NAME injection inhibited the effects of stress. Administration of this drug also resulted in a significant decrease in immobilization time in non-stressed animals [two-way analysis, intragroup: L-NAME effect: F(7,45)=3.27, P<0.001, stress: F(1,45)=4.12, P<0.001, L-NAME vs. stress: F(7,45)=2.3.78, P<0.001], (Figure 4B).

## 4. Discussion

In the current study, maintenance of anxiety and depression in the stressed rats and possible involvement of BLA nitric oxide in this regard was studied. Although chronic stress-induced anxiety and depression are studied, the impact of BLA nitric oxide in maintenance of these consequences is not well understood.

The current study data indicated that stress could induce anxiety and depression in rats 6–8 days after stress termination. It should be mentioned that stress can stimulate brain stress pathways, e.g. the HPA and SA systems and increase blood glucocorticoid hormones as well as adrenaline and noradrenaline (respectively) levels (McEwen, 2012). It is shown that glucocorticoid hormones increment leads to structural and functional changes (remodeling) in the several areas of the brain including prefrontal cortex, hippocampus, and BLA (McEwen, Nasca, & Gray, 2016), which in fact can affect the performance of these areas, including memory and learning, emotions, and decision making as the most adverse effects of chronic stress (McEwen et al., 2016).

Results of the current study showed that the effect of stress was sustained, and can be tracked both in hippocampus and the amygdala after 6–8 days; as it was shown by more pronounced anxiety and depression in the stressed rats. Maintenance of stress effects in the brain is one of the main health problems in societies and some mental illnesses including anxiety, depression, and bipolar disorder (Pacak & Palkovits, 2010). Side effects of the stress maintenance including anxiety and depression are thought to be related to the effects of stress hormones on hippocampal and amygdala neurons (Hunter & McEwen, 2013; Lupien, McEwen, Gunnar, & Heim, 2009). In this regard, a previous study showed that only one time stress immobilization in mouse causes long-lasting morphological changes in the BLA neurons that can be traced for 10 days. In addition, previous studies revealed that chronic restraint stress can induce visible morphological changes in the CA1, CA3, and dentate gyrus areas in the hippocampus (McEwen et al., 2012; Ponomarev, Rau, Eger, Harris, & Fanselow, 2010; Belujon & Grace, 2011). Interestingly, it was observed that these morphological changes co-occurred with depression.

The current study showed that administration of L-arginine as NO precursor and L-NAME as NO synthase inhibitor, inhibited stress-induced anxiety in the animals. This result seems controversial, since both stimulation and inhibition of NO synthesis show similar effects. Moreover, these effects were not dose-dependent. It should be noted that researches show that NO can easily move in the cell’s cytoplasm after synthesis and can spread up to a few microns in the space around the cell. Since this molecule is a free radical, it should be considered that it can attach to the various biomolecule species in the cytoplasm, cell membrane, and also intercellular fluid, and nitrosylate them (Garthwaite & Boulton, 1995). Nitrosylation of biomolecules is an important factor in changing their function (Garthwaite & Boulton, 1995). Since NO does it without special purpose, hence, any macromolecules or biomolecules may be targeted. Therefore, it is difficult to exactly predict the results of NOS activity and/or inhibition.

It is also shown that NO can stimulate the release of various neurotransmitters including dopamine, glutamate, GABA, and acetylcholine (Garthwaite & Boulton, 1995). In this regard and given that glutamate and GABAergic mechanisms are important in the amygdala (Rainnie, Asprodini, & Shinnick-Gallagher, 1991a, b), it may be concluded that the interaction between NO and these two neurotransmitters leads to the above responses. On the other hand, NO is known as a retrograde neurotransmitter that can be produced in post-synaptic cells, where it stimulates the release of glutamate from the presynaptic cell retrogradely. Therefore, one may conclude that by NO manipulation, indirectly, the normal function of glutamate (and may be GABA) in the BLA region is affected. The fourth mechanism by which NO can interfere with stress effect in the BLA is that NO can increase the activity of the guanylate cyclase enzyme in the target neurons (Schafe et al., 2005; Lange, Doengi, Lesting, Pape, & Jungling, 2012).

Guanylate cyclase enzyme increases the production of cGMP, and acts as a secondary mediator in different regions of the brain (Stamler, Lamas, & Fang, 2001). However, there is no data considering the effect of stress on this enzyme in the BLA neurons, and there is no discussion about possible involvement of this enzyme in the current study results. According to these mechanisms, it is not surprising that the responses associated with increasing NO and those of the inhibition of its production are similar. Interestingly, L-arginine and L-NAME in non-stressed animals also produced anti-anxiolytic effects and this effect was more pronounced in L-NAME.

It was observed in previous studies that NOS enzyme inhibition in other regions of the brain such as the dorsal hippocampus can lead to anxiety reduction in mice (Piri, Nasehi, Asgariyan, & Zarrindast, 2012). However, in previous studies, the decrease in motor activity was not statistically significant and the doses used in the current study were different (Piri et al., 2012). Previous research showed that both pyramidal and inter neurons in the BLA contained abundant amounts of neuronal NOS [nNOS] (Usunoff, Itzev, Rolfs, Schmitt, & Wree, 2006). Activation of this enzyme due to the entry of calcium into the above cells, which occurs during glutamate system activity, is one of the most important responses of these neurons (Garthwaite & Boulton, 1995). It is therefore not surprising that nNOS inhibition, or increasing its production by the excess of NO precursor (L-arginine) can interfere with the function of this enzyme (Garthwaite & Boulton, 1995).

According to the role of glutamate system in the mediation of stress effects (Popoli, Yan, McEwen, & Sanacora, 2012), it is not surprising either that nNOS may be affected by stress hormones and nNOS gene, and protein expression also increase in the BLA (Lange et al., 2012). However, it may be proposed that since no specific nNOS inhibitor was employed, it is not easy to conclude that nNOS is the responsible enzyme in the current observation.

The discrepancy may be resolved by considering the fact that other types of the NOS are present in the BLA neurons in a much less amount than that of nNOS (Lange et al., 2012), therefore, it can be concluded that the effects observed in the current study at least may belong to nNOS activity. In a conclusion, the current study data in this part of the experiments were in agreement with the previous studies and confirmed that any changes in the BLA NOS activity can inhibit the effects of stress in the induction of anxiety. The current study did not investigate the expression of NOS, and this could be an important point in future research.

In the next part of the current experiments, stress induced depression and this effect was still present in rats after eight days; as in the case of anxiety, administration of L-arginine and L-NAME prior to stress, inhibited the effects of stress on depression. sIt is observed that the effect of stress on the induction of depression is attributed to the effect of stress hormones on the hippocampus (McEwen, 2000). Many studies show the reduction of dendritic arborization, cell body size, and synaptic communication in the neurons located in the CA1 and CA3 regions in stressed animals or in animals receiving high doses of glucocorticoids (McEwen, 2000), which in fact resulted in verbal, spatial, and emotional memory defect as well as reduction of the inhibitory effect of the hippocampus on the periventricular nucleus of the hypothalamus (McEwen & Sapolsky, 1995). The same mechanisms also could be considered to be functional in the current study.

Results of the current study showed that administration of both L-arginine and L-NAME within BLA can inhibit the effect of stress on induction of depression. It is interesting that, as with anxiety, this inhibition was also dose-independent and can be applied to both drugs. The current study data indicated that inhibition of NOS in the BLA can interact with the effects of stress on the hippocampus, which is the main area involved in depression (McEwen & Sapolsky, 1995) and may explain the presence of an association between BLA and hippocampus.

According to the mechanisms described for NO performance, it is not surprising that in the current study the same responses were observed from L-arginine and L-NAME. Administration of L-arginine and L-NAME within BLA in non-stressed animals reduces their immobility time (reduction of depression as compared with normal animals). It can be concluded that NOS in the BLA may play an important role in depression. Also any of all the four mechanisms mentioned above may be involved in the results.

In conclusion, according to the current study findings, it is important that NO in BLA can reduce anxiety and depression and can inhibit the stress effect on anxiety and depression. Therefore, NO in the BLA is an important modification both in physiological (stress-free) and in the response to stress in rats, which may be true in humans.
